# Assessment of the effect of the SLC5A2 gene on eGFR: a Mendelian randomization study of drug targets for the nephroprotective effect of sodium-glucose cotransporter protein 2 inhibition

**DOI:** 10.3389/fendo.2024.1418575

**Published:** 2024-08-29

**Authors:** Gailing Liu

**Affiliations:** Department of Nephrology, People’s Hospital of Zhengzhou University, He’nan Provincial People’s Hospital, He’nan Provincial Key Laboratory of Kidney Disease and Immunology, Zhengzhou, China

**Keywords:** sodium-glucose cotransporter protein 2 (SGLT2) inhibition, *SLC5A2*, eGFR, drug target Mendelian randomization, insulin resistance

## Abstract

**Aim:**

Sodium-glucose cotransporter protein 2 (SGLT2) inhibitors have been shown to have renoprotective effects in clinical studies. For further validation in terms of genetic variation, drug-targeted Mendelian randomization (MR) was used to investigate the causal role of SGLT2 inhibition on eGFR effects.

**Methods:**

Genetic variants representing SGLT2 inhibition were selected as instrumental variables. Drug target Mendelian randomization analysis was used to investigate the relationship between SGLT2 inhibitors and eGFR. The IVW method was used as the primary analysis method. As a sensitivity analysis, GWAS pooled data from another CKDGen consortium was used to validate the findings.

**Results:**

MR results showed that hemoglobin A1c (HbA1c) levels, regulated by the *SLC5A2* gene, were negatively correlated with eGFR (IVW β -0.038, 95% CI -0.061 to -0.015, P = 0.001 for multi-ancestry populations; IVW β -0.053, 95% CI -0.077 to -0.028, P = 2.45E-05 for populations of European ancestry). This suggests that a 1-SD increase in HbA1c levels, regulated by the SLC5A2 gene, is associated with decreased eGFR. Mimicking pharmacological inhibition by lowering HbA1c per 1-SD unit through SGLT2 inhibition reduces the risk of eGFR decline, demonstrating a renoprotective effect of SGLT2 inhibitors. HbA1c, regulated by the *SLC5A2* gene, was negatively correlated with eGFR in both validation datasets (IVW β -0.027, 95% CI -0.046 to -0.007, P=0.007 for multi-ancestry populations, and IVW β -0.031, 95% CI -0.050 to -0.011, P=0.002 for populations of European origin).

**Conclusions:**

The results of this study indicate that the *SLC5A2* gene is causally associated with eGFR. Inhibition of *SLC5A2* gene expression was linked to higher eGFR. The renoprotective mechanism of SGLT2 inhibitors was verified from the perspective of genetic variation.

## 1 Introduction

Chronic Kidney Disease (CKD) is a disease that seriously jeopardizes human health. In recent years, cardiorenal protection studies have made significant progress in patients with CKD, and the introduction of sodium-glucose cotransporter protein 2 (SGLT2) inhibitors in particular has provided an important new tool in the treatment of CKD (1, 2). These inhibitors target SGLT2, which is responsible for the reabsorption of glucose and sodium in the proximal tubules of the kidneys. SGLT2 inhibitors lower glucose levels and blood pressure by lowering the renal threshold for glucose excretion and by promoting negative salt and water homeostasis. They also have an effect on glomerular hemodynamics, leading to a decrease in glomerular filtration rate, which is reversible when treatment is stopped. This phenomenon is known as “glomerular feedback”. In type 2 diabetes mellitus (T2DM), SGLT2 inhibitors affect glomerular ultrafiltration by decreasing vascular resistance and inducing vasodilation. In addition, these inhibitors reduce fibrosis in the proximal tubule by decreasing energy expenditure and inhibiting the mammalian target of rapamycin (mTOR) pathway (3). Another growing area of research is the ability of SGLT2 inhibitors to induce a fasting state (4). All of these effects reduce cellular stress and inflammation in cardiac and renal tissues, which is thought to be the pathophysiologic reason for the beneficial effects of SGLT2 inhibitors on cardiac and renal function (1).

SGLT2 inhibitors, including canagliflozin, dapagliflozin, and empagliflozin, are widely approved antihyperglycemic agents that improve insulin resistance (IR) (5), and are used to reduce glycemia and cardiovascular risk in T2DM patients (6). Large clinical trials provide compelling evidence to support their beneficial effects on major adverse cardiovascular events, hospitalization for heart failure (7–11) and renal outcomes (12, 13). However, studies on genetic variants related to SGLT2 inhibitors’ renoprotective effects are limited. The *SLC5A2* gene encodes SGLT2, crucial for renal glucose reabsorption. Despite promising results in clinical studies, challenges such as variable patient responses and long-term effects remain. Addressing these challenges is essential to optimize clinical application.

In the field of precision medicine, finding effective molecular targets is a key task. Traditional methods to study the mechanism of action of target proteins through pharmacology are inefficient and time-consuming. The drug target Mendelian randomization (MR) has emerged as a novel and powerful research method. Novel methods such as gene co-localization and gene variants associated with drug target mRNA expression (expression quantitative trait loci (*eQTL*)) can be used to create MR tool variables for drug exposure (14). The *eQTL* is a type of expressed quantitative trait locus, which can be classified into *cis-eQTL* and *trans-eQTL* by analyzing the relationship between the amount of gene expression and the locus. The former regulates genes that are closer together, while the latter regulates genes that are farther apart. Drug target MR utilizes genetic instrumental variables to explore the causal relationship between molecules and diseases. This approach is based on the downstream products of target proteins, using Single Nucleotide Polymorphisms (SNPs) near the coding genes of the target proteins that have significant effects on these downstream products (protein Quantitative Trait Loci (*pQTL*) or *eQTL*) as the instrumental variables. The concentration of the downstream products of the target proteins serves as the exposure, while disease serves as the endpoint. MR is then performed to verify the effect of the target proteins on the studied diseases, providing new ideas for drug development and effect prediction. This strategy can be used to investigate the biological mechanism of the effect of SGLT2 inhibition on eGFR.

Given that the genetic mechanisms of SGLT2 inhibition in CKD protection are poorly understood, we hypothesized that SGLT2 inhibitors may be causally related to eGFR. Therefore, our study was designed to assess the genetic causal effect of SGLT2 inhibition on eGFR. We performed a two-sample MR analysis using genetic variants associated with *SLC5A2* gene expression and hemoglobin A1c (HbA1c) levels as instrumental variables. This helps to expand our understanding of the mechanisms of SGLT2 inhibitors in renal protection and provides evidence for this.

## 2 Methods

### 2.1 Study design

**Figure 1:**
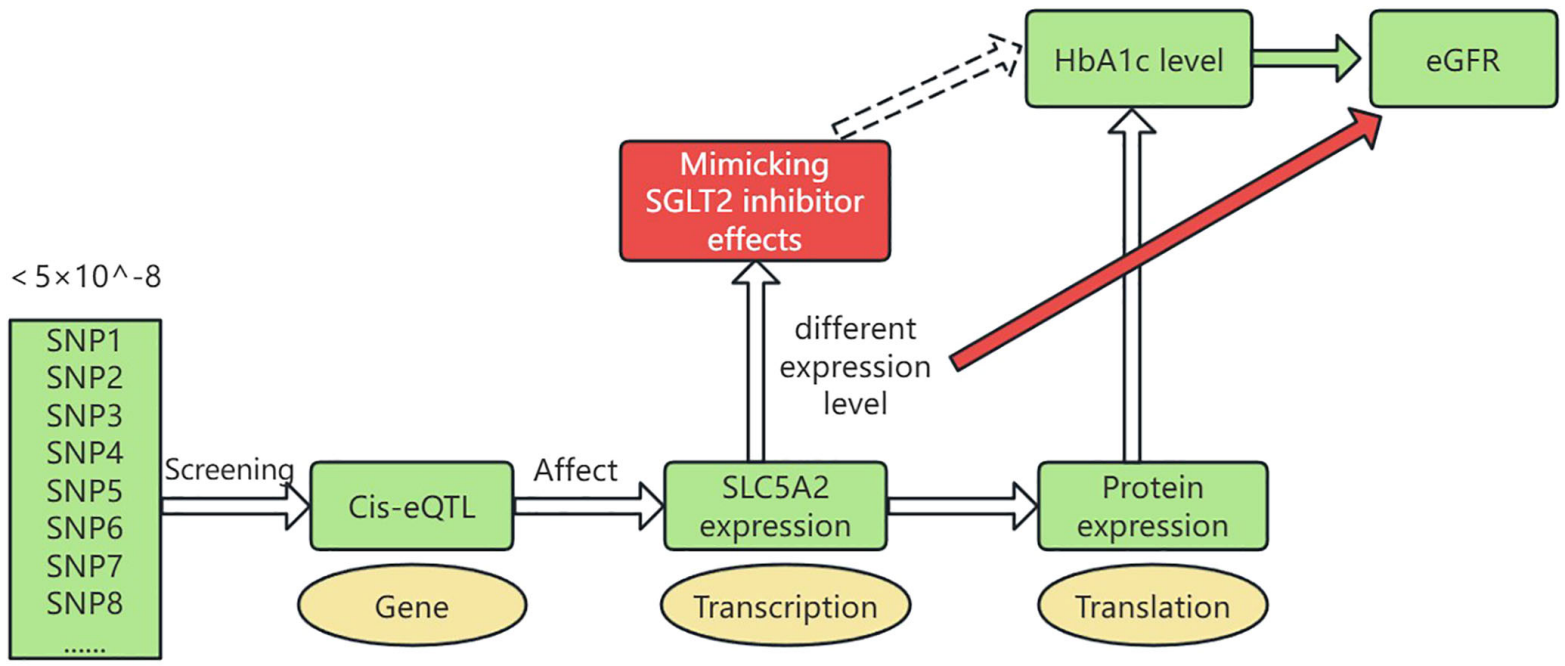
Drug-targeted MR study design. SGLT2, Sodium–glucose cotransporter 2; HbA1c, hemoglobin A1c; eGFR, estimated Glomerular Filtration Rate; cis-eQTL, cis-expression Quantitative Trait Locus. Genetic variants representing SLC5A2 gene expression levels were selected by screening cis-eQTLs with a genotype frequency of less than 5×10^-8. These variants influence SLC5A2 gene expression at the RNA transcription level. The expression further affects protein levels, which in turn influence HbA1c levels. Thus, different expression levels of the SLC5A2 gene can mimic the therapeutic effects of SGLT2 inhibitors (glucose-lowering effects, reduced HbA1c). We studied the impact of these varying expression levels on eGFR.

**Figure 1** illustrates a schematic of the study design. The study was conducted in the following five steps: 1) Genetic variants representing the effects of SGLT2 inhibition were selected as instrumental variables. 2) Pooled GWAS data for T2DM was selected as a positive control. 3) The outcome factor, eGFR, was selected. 4) MR analyses were performed to estimate the causal effects of SGLT2 inhibition on eGFR.

### 2.2 Data sources

#### 2.2.1 Selection of genetic variants of SGLT2 inhibitors

The process of selecting genetic variants for SGLT2 inhibitors is described in detail in the study of Xu M. et al. (15). It involves four steps: First, genetic variants associated with *SLC5A2* gene expression were selected by Genotype-Tissue Expression (GTEx) (16) and *eQTL*Gen Consortium (17) data; second, the correlation of each *SLC5A2* variant with HbA1c levels was assessed using UK Biobank data (18); then, the causal variants of *SLC5A2* with HbA1c were verified by the co-localization method (10); and finally, six genetic variants closely associated with HbA1c and SGLT2 inhibition were screened for MR analysis by the standard clustering process and F-statistic estimation (**Supplementary Table 1**).

#### 2.2.2 Study outcome

The GWAS data used in this study included the year of publication, sample size, SNP counts, and population information, as detailed in **Table 1**. The outcome GWAS data in this study were extracted from specialized disease databases. GWAS data for T2DM were obtained from the Diabetes Genetics Replication and Meta-analysis (DIAGRAM) consortium, and GWAS data for eGFR were obtained from the CKDGen consortium.

**Table 1:** Description of GWAS data sources.

Variable	Phenotype	Sample size	Ancestry	Cohort/Consortium	First author	Year	PubMed ID
Exposure	SLC5A2	344182	European	UK Biobank	NA	2018	NA
Positive control	T2DM	673025	Multi-ancestry	DIAGRAM	Mahajan A	2022	35551307
Outcome-ALL	eGFR	1,046,070	Multi-ancestry	CKDGen	Wuttke M	2019	31152163
Outcome-EA	eGFR	1,046,070	European	CKDGen	Wuttke M	2019	31152163
Validation dataset-ALL	eGFR	1201929	Multi-ancestry	CKDGen, UK Biobank	Stanzick KJ	2021	34272381
Validation dataset-EA	eGFR	1004040	European	CKDGen, UK Biobank	Stanzick KJ	2021	34272381

GWAS, Genome-Wide Association Study; T2DM, Diabetes Mellitus Type 2; eGFR, estimated Glomerular Filtration Rate; DIAGRAM, Diabetes Genetics Replication and Meta- analysis; CKDGen, Chronic Kidney Disease Gen; ALL, Multi-ancestry population; EA, European population.

NA, Not Available.

##### 2.2.2.1 Positive control data

Positive control MR analysis justifies the genetic tool of a drug by demonstrating the expected effect on an outcome that has an established causal relationship with the target drug (19). SGLT2 inhibitors are used as a glucose-lowering drug with the intended indication of T2DM. Therefore, T2DM GWAS pooled data was selected as a positive control. The T2DM data in this study was obtained from one of the largest diabetes case-control studies published in 2022 in the DIAGRAM consortium. This study was conducted by Mahajan et al. The GWAS meta-analysis included 22 GWAS studies involving 180,834 patients with T2DM and 492,191 controls, as well as five lineage groups (51.1% of European ancestry) (20).

##### 2.2.2.2 Study outcome data

Genetic associations for eGFR were obtained from a meta-analysis of publicly available CKDGen studies (21). All participants were of European ancestry, with a mean age of 54 years. All genetic correlations were adjusted for sex, age, study center, genetic rationale, components, correlations, and other study-specific characteristics (22). To confirm the robustness of the results of this study, we performed MR analysis using eGFR meta-analysis GWAS data from a diverse ancestry cohort (n ≥ 61 studies) and from a European ancestry cohort (n ≥ 42 studies). Detailed information is provided in **Table 1**.

##### 2.2.2.3 Validation of outcome data

In addition, as a sensitivity analysis, the results of this study were validated using GWAS pooled data from another CKDGen meta-analysis using eGFR GWAS data from Stanzick et al, which is publicly accessible on the official website of CKDGen (23). Also, this data has been integrated into the GWAS Catalog database (GCST90103633, GCST90103634) (**Table 1**).

The GWAS summary statistics used in this study can be found in the IEU OpenGWAS project (https://gwas.mrcieu.ac.uk/), the GTEx portal (https://www.gtexportal.org/), the *eQTL*Gen consortium (https://eqtlgen.org/), the DIAGRAM consortium (https://diagram-consortium.org/), CKDGen (https://ckdgen.imbi.uni-freiburg.de/), UKB Research (https://www.nealelab.is/uk-biobank, ukb-d-30750_irnt), and the GWAS Catalog (https://www.ebi.ac.uk/gwas/downloads/summary-statistics, GCST008058, GCST008064, GCST90103633, GCST90103634).

### 2.3 Statistical analysis

In the first place, genetic variant screening for SGLT2 inhibitors applied a generalized inverse variance weighting (IVW) method to consider the correlation of six predictors. MR effects were estimated by the LD matrix and IVW and MR-Egger methods to improve the efficiency and stability of the analysis (24). Subsequently, in MR analysis, the IVW method was used as the primary method to explore the effect of SGLT2 inhibition on the positive control T2DM and the study outcome eGFR (19). Various methods (including MR-Egger, weighted median, simple, and weighted models) were applied to test the reliability and stability of the results. The Strengthening the Reporting of Observational Studies in Epidemiology-Mendelian Randomization (STROBE-MR) guidelines were applied to guide the design of this study and the writing of the results (19). All statistical analyses were performed using the “Two-sample MR” (version 0.5.6) and “MR” (version 0.5.1) packages in the R software (version 4.3.2) environment. To more accurately interpret causality, we set the level of statistical significance to the Bonferroni-corrected level, that is, P < 0.0083. All MR analyses were performed using the Mendelian randomization R package and the TwoSampleMR R package (github.com/MRCIEU/TwoSampleMR). We plotted the results as forest plots using code from the ggplot 2 package in R.

As a sensitivity analysis, we applied an independent validation with a separate dataset. The validation data came from GWAS pooled data from another CKDGen meta-analysis, details of which are shown in **Table 1**.

To control for heterogeneity, we applied the MR multivalent residuals and outliers (MR-PRESSO) method. To test potential MR hypotheses, such as assessing the effect of directional pleiotropy, we applied the generalized MR-Egger regression method. Both tests of pleiotropy using the MR-Egger intercept term and tests of heterogeneity among predictors (Cochran Q statistic for IVW and global test for MR-PRESSO) were used to quantify the level of pleiotropy in the MR analysis. Compared to the MR-Egger, the MR-PRESSO method has higher accuracy for identifying levels of multivalence and outliers (25). The stability of outliers and results was also tested using the leave-one-out sensitivity analysis method, funnel plots, and scatter plots.

For a more rigorous interpretation of causality, we also used the Bonferroni correction based on the number of SNPs analyzed in the final MR. We used the significance level after performing the Bonferroni correction, which was set at 0.0083 (0.05 divided by 6 SNPs), to control for the rate of false discovery of multiple comparisons. In each independent SNP analysis, we compared the P value to 0.0083 to determine whether statistical significance existed.

## 3 Results

### 3.1 Effect of positive control-*SLC5A2* on T2DM

To conduct a positive control, T2DM GWAS data were selected as outcome data and analyzed using MR with *SLC5A2*. As shown in **Table 2**, the MR results indicated that a 1-SD increase in HbA1c regulated by *SLC5A2* was associated with an 83% increase in the risk of T2DM (IVW OR 1.83, 95% CI: 1.180-2.846, p = 0.007). This result effectively mimics the pharmacological inhibition mechanism of SGLT2 inhibitors, demonstrating that SGLT2 inhibition reduces the risk of T2DM. SGLT2 inhibitors, as glucose-lowering drugs, have an established causal relationship with T2DM, and the results of the positive control analysis were consistent with the expected outcomes. This suggests that SGLT2 inhibition decreases the risk of T2DM and demonstrates that the target gene, *SLC5A2*, can serve as a genetic tool for the drug target of SGLT2 inhibitors.

**Table 2:** MR estimation of the effect of SLC5A2 on T2DM and eGFR.

Exposure	Group	Outcome	Method	nSNP	β (95% CI)	SE	OR (95% CI)	P-value	P_h_	P_intercept_
SLC5A2	Positive control	T2DM	MR Egger	6	-1.456(-3.641~0.729)	1.115	0.233(0.026~2.072)	0.262	0.913	0.132
			Weighted median	6	0.512(-0.080~1.103)	0.302	1.668(0.941~2.955)	0.090		
			IVW	6	0.606(0.165~1.046)	0.225	1.832(1.180~2.846)	0.007	0.474	
	Outcome-ALL	eGFR	MR Egger	6	0.049(-0.051~0.149)	0.051		0.389	0.476	0.157
			Weighted median	6	-0.028(-0.055~-0.002)	0.013		0.035		
			IVW	6	-0.038(-0.061~-0.015)	0.012		0.001	0.258	
	Outcome-EA	eGFR	MR Egger	6	-0.006(-0.124~0.113)	0.060		0.932	0.412	0.471
			Weighted median	6	-0.064(-0.098~-0.030)	0.017		2.507E-04		
			IVW	6	-0.053(-0.077~-0.028)	0.012		2.451E-05	0.468	
	Validation dataset-ALL	eGFR	MR Egger	6	0.027(-0.072~0.127)	0.051		0.620	0.969	0.340
			Weighted median	6	-0.025(-0.049~-0.001)	0.012		0.041		
			IVW	6	-0.027(-0.046~-0.007)	0.010		0.007	0.887	
	Validation dataset-EA	eGFR	MR Egger	6	-0.009(-0.109~0.091)	0.051		0.867	0.948	0.688
			Weighted median	6	-0.031(-0.055~-0.006)	0.012		0.014		
			IVW	6	-0.031(-0.050~-0.011)	0.010		0.002	0.969	

T2DM, Diabetes Mellitus Type 2; eGFR, estimated Glomerular Filtration Rate; 95% CI, 95% confidence interval; IVW, Inverse Variance Weighted; nSNP, Number of Single Nucleotide Polymorphism; SE, standard deviation; P_h_, Heterogeneity P-value; P_intercept_, MR-Egger regression intercept P-value; ALL, Multi-ancestry population; EA, European population.

### 3.2 Effect of *SLC5A2* on eGFR

**Figure 2:**
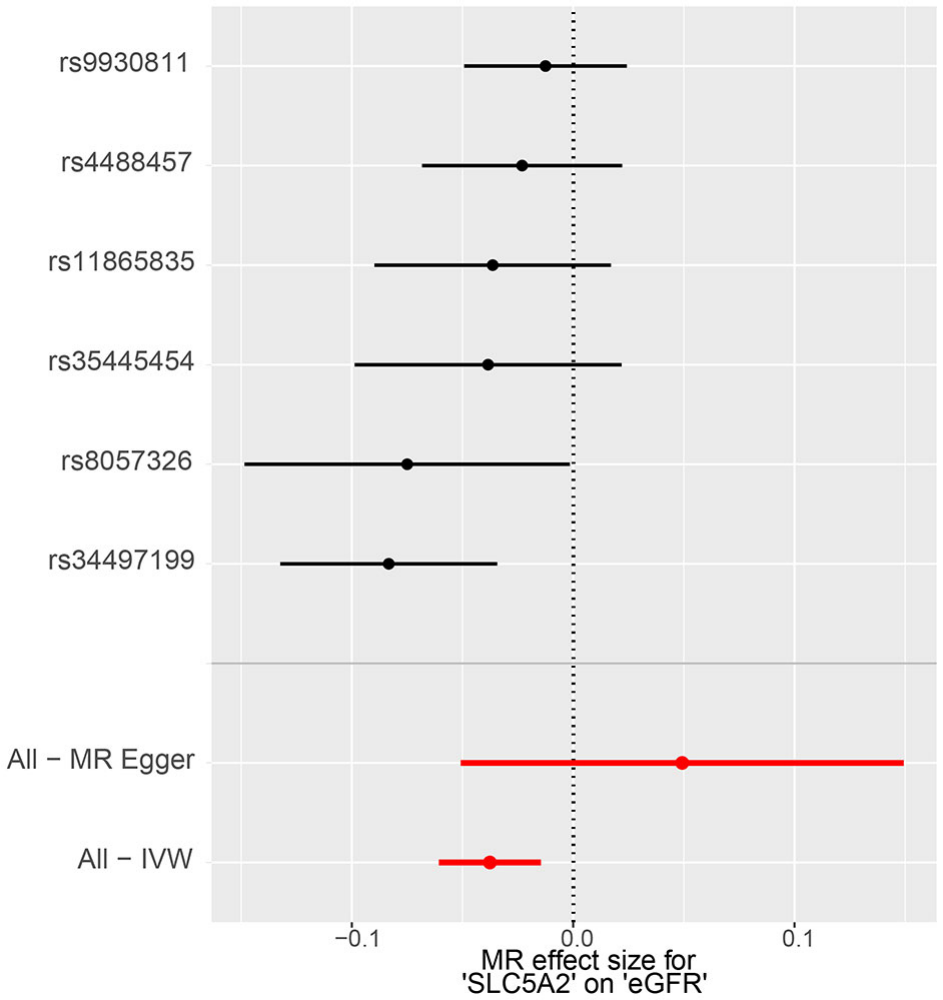
MR forest plot of the effect of SLC5A2 on eGFR in multi-ancestry populations. eGFR, estimated Glomerular Filtration Rate; IVW, Inverse Variance Weighted; MR, Mendelian randomization.

**Figure 3:**
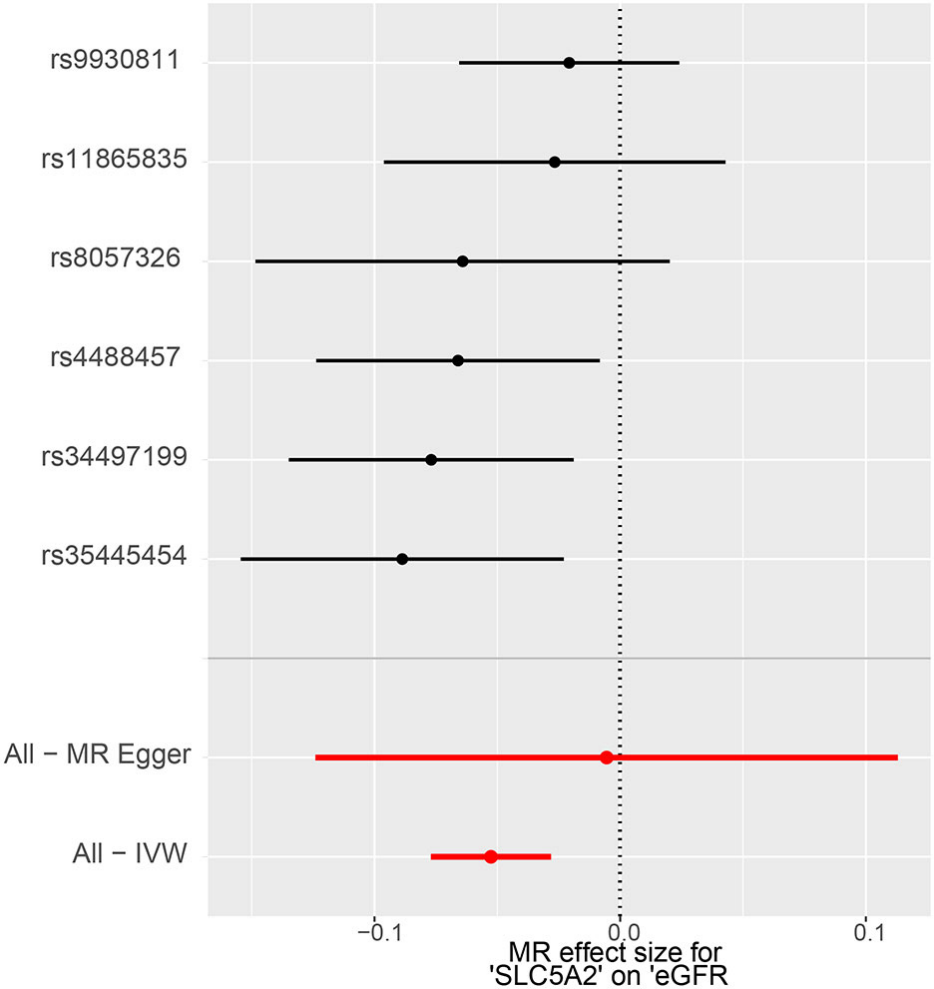
MR forest plot of the effect of SLC5A2 on eGFR in European populations. eGFR, estimated Glomerular Filtration Rate; IVW, Inverse Variance Weighted; MR, Mendelian randomization.

The IVW method showed evidence to support a causal association between *SLC5A2* and eGFR (**Table 2**, **Figures 2**, **3**). As shown in **Table 2**, the MR results indicated that the *SLC5A2* gene, was negatively correlated with eGFR (IVW β -0.038, 95% CI -0.061 to -0.015, P = 0.001 for multi-ancestry populations; IVW β -0.053, 95% CI -0.077 to -0.028, P = 2.45E-05 for populations of European ancestry). This suggests that each 1-SD increase in HbA1c predicted by the SLC5A2 gene is associated with a decrease in eGFR.

Conversion to mimic pharmacological inhibition, that is, lowering genetically predicted HbA1c per 1-SD unit by SGLT2 inhibition, reduces the risk of eGFR decline, which means that SGLT2 inhibitors are demonstrated to have a nephroprotective effect. All of the above P-values were less than 0.083 (Bonferroni-corrected significance level), suggesting statistical significance and robust results.

### 3.3 Validation and sensitivity analysis

First, as a sensitivity analysis, this study was validated using GWAS pooled data from another CKDGen meta-analysis, which yielded consistent findings across both multi-ancestry and European populations (**Table 2**, **Supplementary Figures 1**, **2**). The validation data indicated a negative correlation between HbA1c predicted by the gene SLC5A2 and eGFR. Specifically, for multi-ancestry populations, IVW β was -0.027 (95% CI -0.046 to -0.007, P=0.007), and for populations of European ancestry, IVW β was -0.031 (95% CI -0.050 to -0.011, P=0.002). These results are consistent with the primary study outcomes.

Secondly, as shown in **Table 2**, MR analyses applying the weighted median method in the positive control, primary outcome, and validation outcome groups yielded consistent findings.

Third, the IVW heterogeneity test using the Cochran Q test in each group separately showed that neither the Q statistic nor the P value was significant (P > 0.05), implying that there was no evidence of heterogeneity in the effect of SGLT2 inhibition on eGFR. The test of pleiotropy using the MR-Egger intercept term showed that the P-value for the intercept was greater than 0.05, which implies that horizontal pleiotropy was not shown (**Table 2**).

Fourth, as shown in **Supplementary Figures 3**-**6**, the leave-one-out analysis in the primary outcome data and validation data for both multi-ancestry and European populations indicated that the effect sizes remained on the same side of the reference line 0, demonstrating robust results. This analysis confirms the negative correlation between the SLC5A2 gene and eGFR.

Overall, the results of the MR analysis in this study were robust.

## 4 Discussion

In this study, we performed MR analysis of drug targets and determined the genetic causal effect of SGLT2 inhibitors on eGFR. This finding provides genetic validation of the mechanism of action of SGLT2 inhibitors in improving renal function and delaying CKD progression.

Several large clinical observational or randomized controlled studies have found that canagliflozin, dapagliflozin, and empagliflozin produce a sustained improvement in kidney disease prognosis, including slowing eGFR decline, reducing albuminuria, and lowering the risk of renal failure (1, 13, 26, 27). This improvement is not only seen in T2DM patients, but patients without diabetes can also benefit. Based on extensive evidence of SGLT2 inhibitors in patients with CKD, KDIGO updated its clinical practice guideline in 2022 to recommend SGLT2 inhibitors as preferred therapeutic agents for managing T2DM combined with CKD (2). SGLT2 inhibitors are crucial for managing T2DM combined with atherosclerotic cardiovascular disease (ASCVD). They are the first-line therapeutic agents for patients with T2DM combined with ASCVD, cardiovascular risk factors, heart failure (HF), and CKD. Additionally, SGLT2 inhibitors are recommended for treating patients with HF or CKD who do not have comorbid diabetes (28).

It is worth noting that the currently completed randomized controlled trials of various SGLT2 inhibitors have different requirements for minimum eGFR levels in the enrollment criteria. Moreover, there are differences in the design of clinical studies for different SGLT2 inhibitors, and the respective protocols may be heterogeneous, so the findings of clinical studies corresponding to one drug may not be directly generalizable to other drugs of the same class. Randomized controlled trials are generally costly and lengthy. In contrast, a new research approach in recent years, drug-targeted MR, has significant advantages. MR is a method that utilizes genetic variants closely associated with exposure as a potentially unconfounded tool to infer a causal relationship between exposure and outcome. New approaches, such as genetic co-localization and genetic variation (*eQTL*) associated with drug target mRNA expression, can be used to create MR instrumental variables for use in drug targets (14), which has become a new research hotspot.

Previous MR studies have further explored the benefits of SGLT2 inhibitors. Xu M et al. (15) investigated the role of SGLT2 inhibition in reducing coronary artery disease (CAD) and T2DM. They found that SGLT2 inhibition lowered the risk of both conditions, with choline metabolites partly mediating these effects. Another study focused on HF and identified that SGLT2 inhibition reduced HF risk through anti-inflammatory mechanisms, specifically via the CXCL10 biomarker (29). These studies support our findings and highlight the broader therapeutic potential of SGLT2 inhibitors in managing complex diseases like CKD, T2DM, CAD, and HF.

To the best of our knowledge, few relevant MR studies have explored the genetic causal effect of SGLT2 inhibition on the effects of eGFR. A recently published study by Wang Z et al. investigated the role of circulating metabolites in the renoprotective effects of SGLT2 inhibition, specifically identifying small high-density lipoprotein (HDL) particles as mediators in this process (30). However, our study differs in that it focuses on the direct genetic causal effect of SGLT2 inhibition on eGFR using MR analysis without considering metabolic intermediates. Our MR analysis revealed that genetically predicted SGLT2 inhibition resulted in a reduced risk of eGFR decline. This result supports the association between IR and renal function found in previous observational studies, using genetic data to provide further causal evidence. The EMPA-KIDNEY trial (27), the most extensive international multicenter, randomized, double-blind, placebo-controlled clinical trial in CKD to date, confirmed that empagliflozin treatment significantly reduced the risk of the primary endpoint event, which includes kidney disease progression and cardiovascular death, compared to placebo. An updated subgroup analysis of the EMPA-KIDNEY study confirmed that Engeletin significantly slows the progression of chronic kidney function in a broad population of CKD patients regardless of CKD etiology, diabetes status, eGFR and urinary ACR levels (31, 32). Our findings provide timely and robust evidence for the role of SGLT2 inhibition in reducing CKD risk.

The potential mechanisms linking SLC5A2 inhibition and eGFR may involve reduced expression of SLC5A2, inhibition of SGLT2 receptors, leading to decreased reabsorption of glucose and sodium in renal tubules, which results in osmotic diuresis and lower blood pressure (1). Additionally, these inhibitors may exert anti-inflammatory effects, alleviate renal oxidative stress, and modulate metabolic pathways, collectively supporting renal function (3, 4).

However, there are some limitations to this study. First, this study could only predict the effect of drugs on eGFR by interfering with the encoded proteins (targeting effect). We cannot rule out the possibility that drugs alter eGFR risk through other proteins (off-target effects). Second, our MR analyses estimated SGLT2 inhibition based on targeted reductions in genetically predicted HbA1c levels rather than direct effects of SGLT2 inhibitors. This is based on the assumption that the effect of SGLT2 inhibitors on HbA1c levels is proportional to their overall effect, which may be inconsistent with the actual mechanism of SGLT2 inhibitors. In addition, the drug formulation, dosage, and mode of administration of SGLT2 inhibitors can influence their effects on eGFR. These factors should be taken into account when interpreting our findings. Third, genetic variation reflects the effect of lifelong exposure to a biomarker on outcome and therefore cannot be used to directly estimate the expected impact of short-term pharmacologic changes in that biomarker (33). This means that the effect sizes in our study may not be comparable to those reported in trials or observational studies. Fourth, although the MR-Egger method is dependent on the orientations of SNPs and can influence the outcome of the analysis, our study conducted a pleiotropy test using the MR-Egger intercept term, which showed no evidence of horizontal pleiotropy (P > 0.05). Finally, our analyses were conducted mainly in European populations. Therefore, this limits the generalizability of our findings to other ethnicities. The potential for confounding factors in observational data must also be considered, as they might affect the accuracy and applicability of the results.

Future studies should explore the genetic causal effect of SGLT2 inhibition on eGFR in diverse populations to enhance the generalizability of the findings. Additionally, future research should aim to integrate genetic evidence with experimental and clinical studies to provide a comprehensive understanding of how SGLT2 inhibitors can be leveraged to protect against CKD progression.

## 5 Conclusions

Our results show that *SLC5A2* is negatively correlated with eGFR, identifying a causal role of SGLT2 inhibition with eGFR. By modeling the drug-targeted effects of SGLT2 inhibitors, it was thus concluded that inhibition of *SLC5A2* gene expression was causally associated with higher eGFR. The renoprotective effect of SGLT2 inhibitors was validated from the perspective of genetic variation, and also provided MR evidence for SGLT2 inhibitors as first-line therapeutic agents for CKD patients.

## Data Availability

The datasets presented in this study can be found in online repositories. The names of the repository/repositories and accession number(s) can be found in the article/**Supplementary material**.
