# Supplementary material for: Assessment of the effect of the SLC5A2 gene on eGFR: a Mendelian randomization study of drug targets for the nephroprotective effect of sodium-glucose cotransporter protein 2 inhibition
Source: Front Endocrinol (Lausanne). 2024 Aug 29;15:1418575. doi: 10.3389/fendo.2024.1418575 (PMC11390543; doi:10.3389/fendo.2024.1418575)
Supplement: Supplementary file 1 [file DataSheet1.docx]

**Supplementary material**

**Supplementary TABLE 1** Genetic variants selected as instrumental variables for SGLT2 inhibitors.

| **Tissue** | **Gene**  **name** | **ENSG.ID** | **SNP** | **Chr** | **Position** | **Effect_**  **allele** | **Other_**  **allele** | **EAF** | **Beta** | **Se** | **P** | **N** | **F-**  **statistics** |
| --- | --- | --- | --- | --- | --- | --- | --- | --- | --- | --- | --- | --- | --- |
| Adipose_Visceral_  Omentum | SLC5A2 | ENSG0000  0140675.12 | rs4488457 | 16 | 31659189 | G | T | 0.712 | -0.013 | 0.003 | 2.90E-07 | 344182 | NR |
| Artery_  Coronary | SLC5A2 | ENSG0000  0140675.12 | rs8057326 | 16 | 31524123 | C | T | 0.523 | -0.008 | 0.002 | 2.80E-04 | 344182 | NR |
| Colon_  Transverse | SLC5A2 | ENSG0000  0140675.12 | rs11865835 | 16 | 31509816 | C | T | 0.284 | -0.011 | 0.003 | 1.34E-05 | 344182 | NR |
| Nerve_  Tibial | SLC5A2 | ENSG0000  0140675.12 | rs9930811 | 16 | 31400360 | G | A | 0.365 | -0.016 | 0.002 | 8.69E-12 | 344182 | NR |
| Stomach | SLC5A2 | ENSG0000  0140675.12 | rs34497199 | 16 | 31551332 | T | C | 0.475 | -0.012 | 0.002 | 5.98E-07 | 344182 | NR |
| Uterus | SLC5A2 | ENSG0000  0140675.12 | rs35445454 | 16 | 31699326 | T | C | 0.344 | -0.013 | 0.002 | 1.24E-07 | 344182 | 24.066 |

**SUPPLEMENTARY FIGURE 1** | MR forest plot of the effect of SLC5A2 on eGFR in multi-ancestry populations from validation data.

**SUPPLEMENTARY FIGURE 2** | MR forest plot of the effect of SLC5A2 on eGFR in European populations from validation data.

**SUPPLEMENTARY FIGURE 3** | MR leave-one-out plot of the effect of SLC5A2 on eGFR in multi-ancestry populations.

**SUPPLEMENTARY FIGURE 4** | MR leave-one-out plot of the effect of SLC5A2 on eGFR in European populations.

**SUPPLEMENTARY FIGURE 5** | MR leave-one-out plot of the effect of SLC5A2 on eGFR in multi-ancestry populations from validation data.

**SUPPLEMENTARY FIGURE 6** | MR leave-one-out plot of the effect of SLC5A2 on eGFR in European populations from validation data.
