# Supplementary figures and images for: Assessment of the effect of the SLC5A2 gene on eGFR: a Mendelian randomization study of drug targets for the nephroprotective effect of sodium-glucose cotransporter protein 2 inhibition
Source: Front Endocrinol (Lausanne). 2024 Aug 29;15:1418575. doi: 10.3389/fendo.2024.1418575 (PMC11390543; doi:10.3389/fendo.2024.1418575)

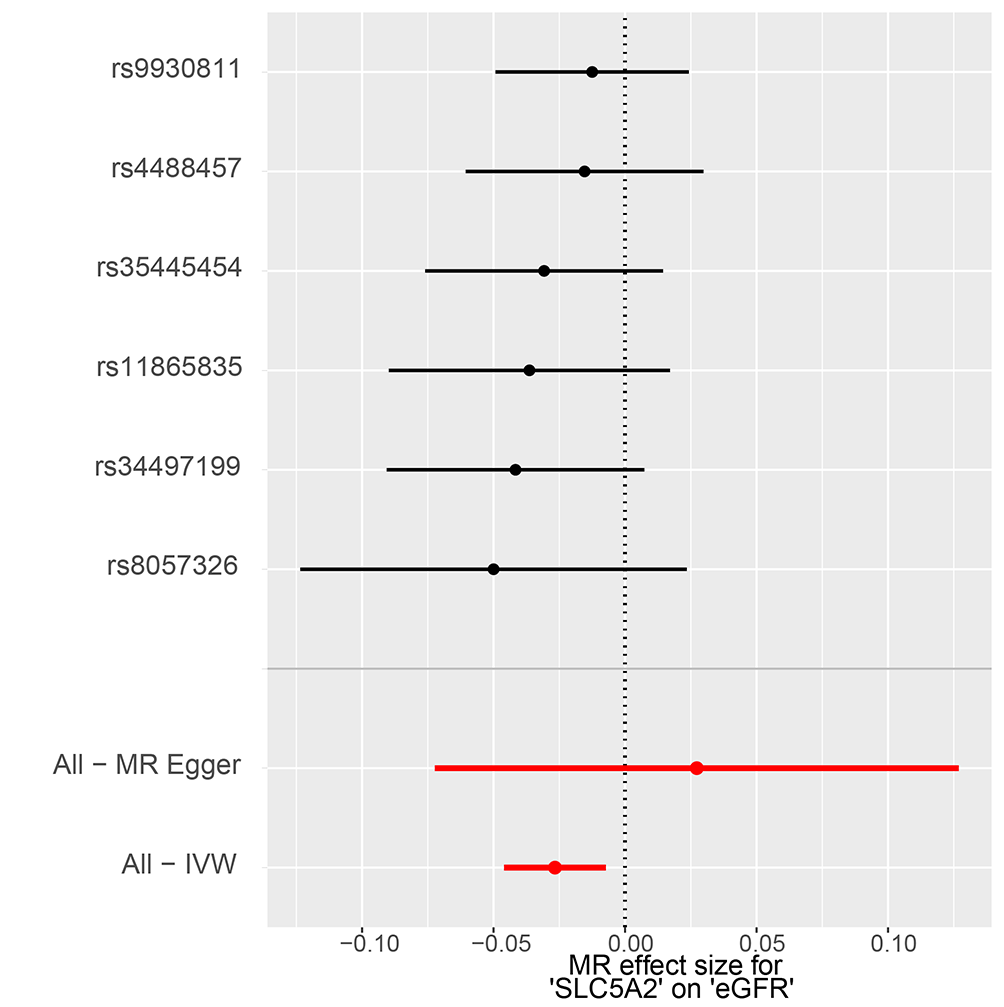

Supplement: Supplementary file 2 [file Image1.tif]

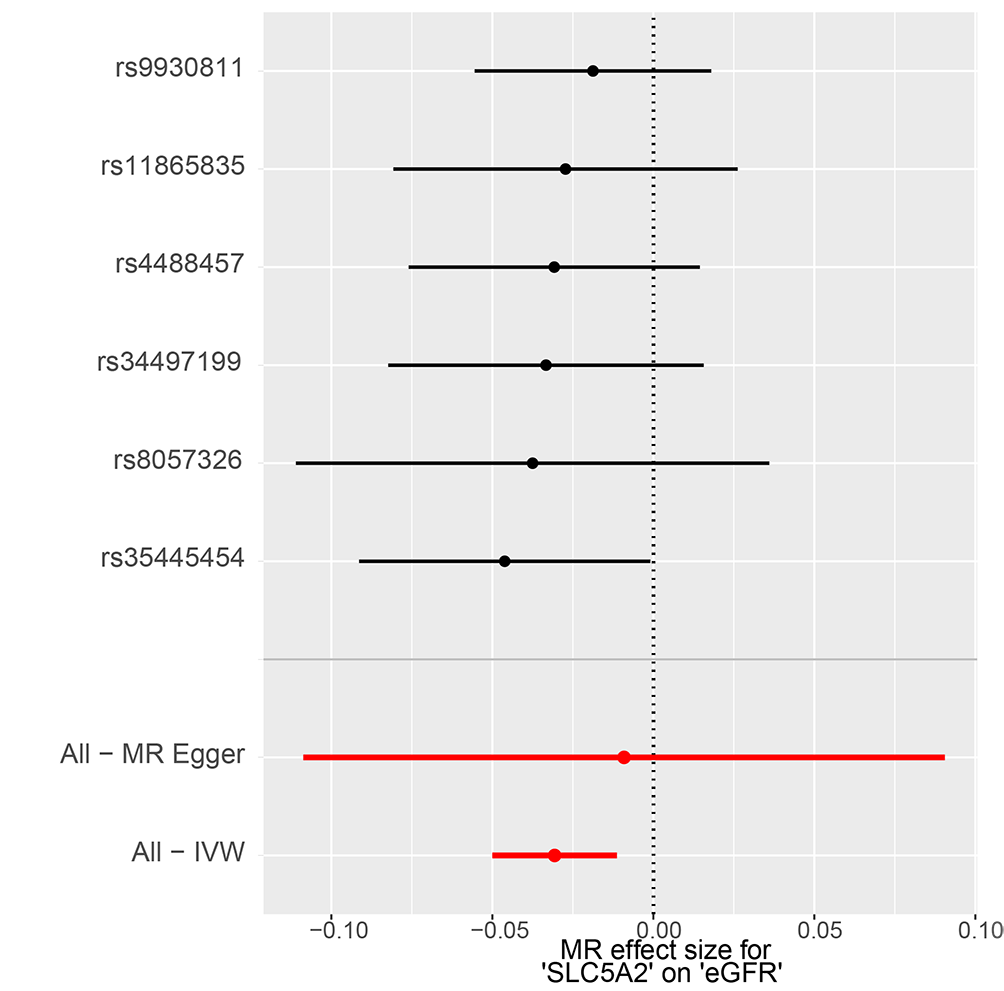

Supplement: Supplementary file 3 [file Image2.tif]

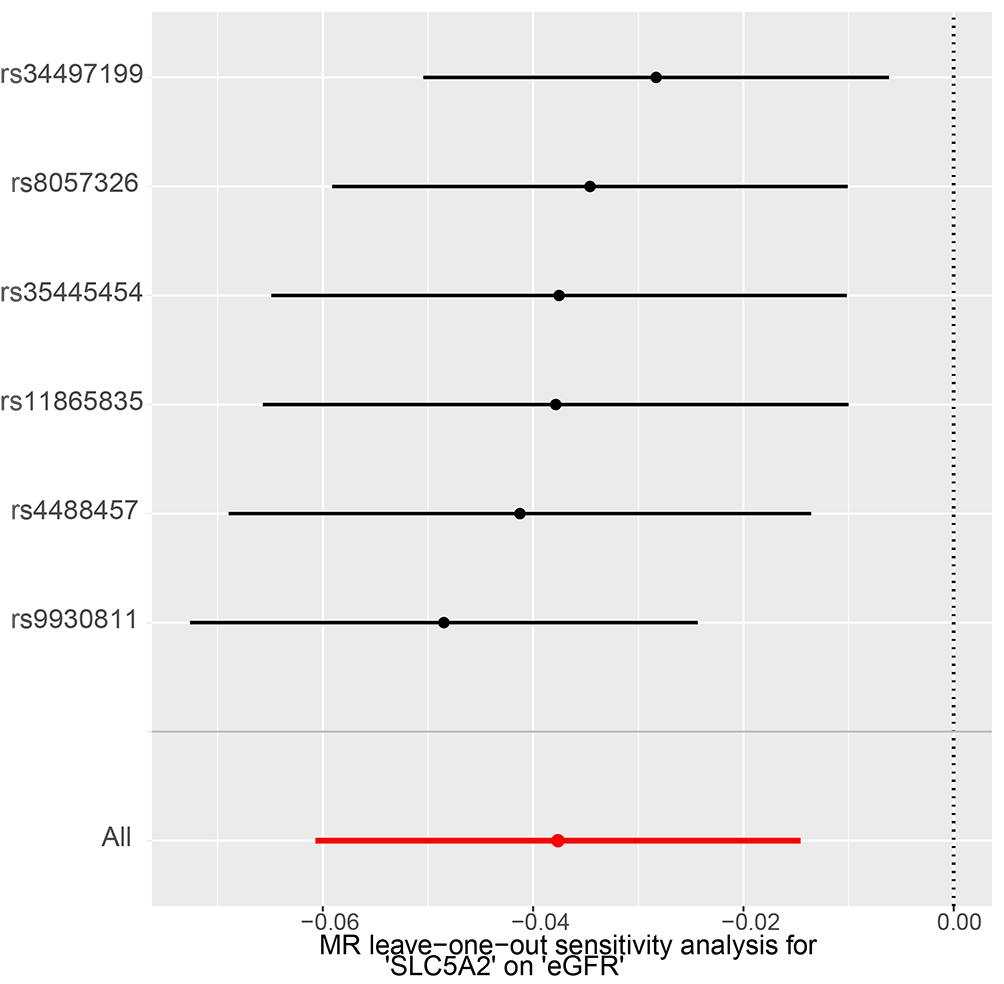

Supplement: Supplementary file 4 [file Image3.tif]

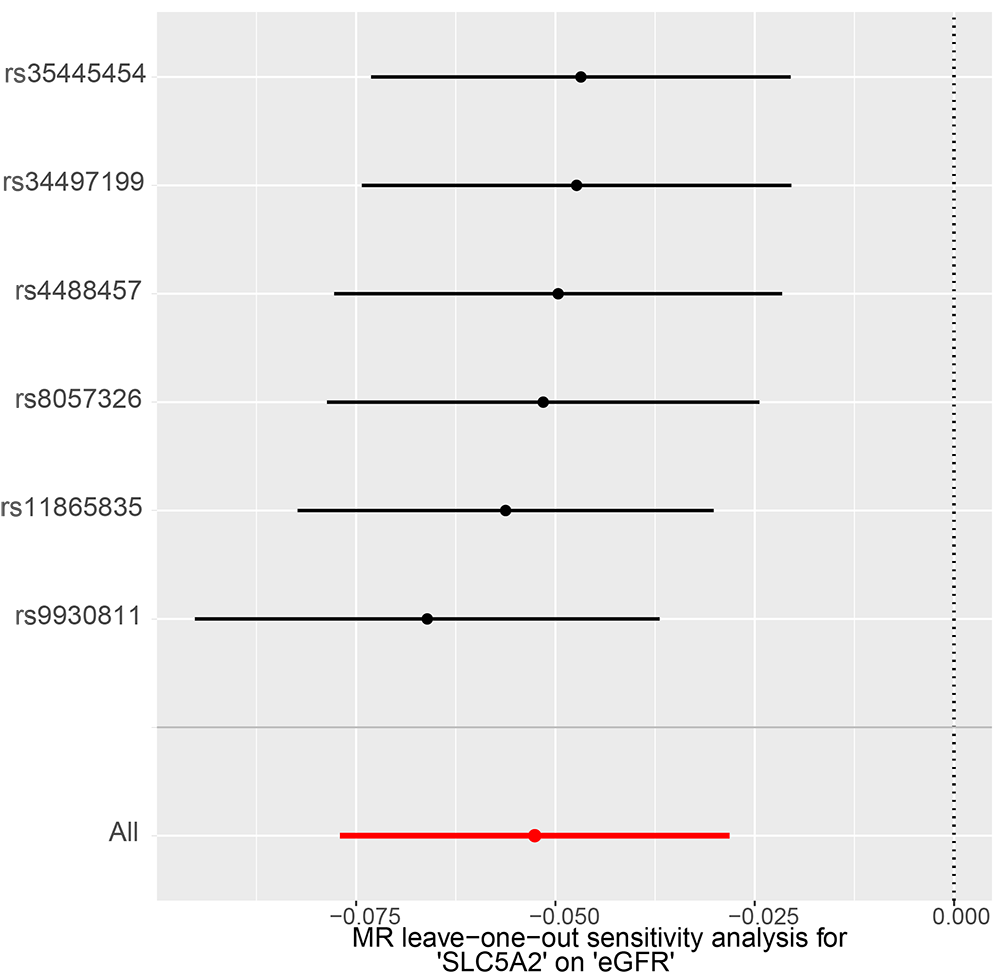

Supplement: Supplementary file 5 [file Image4.tif]

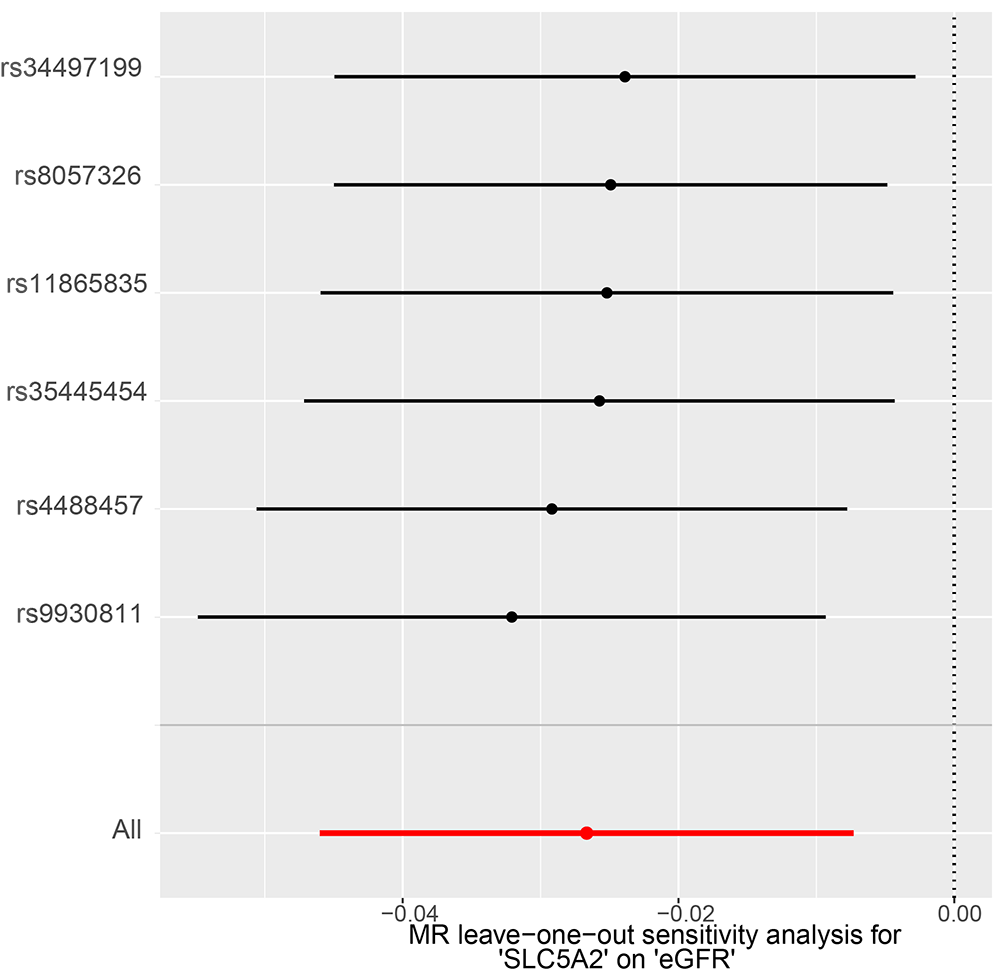

Supplement: Supplementary file 6 [file Image5.tif]

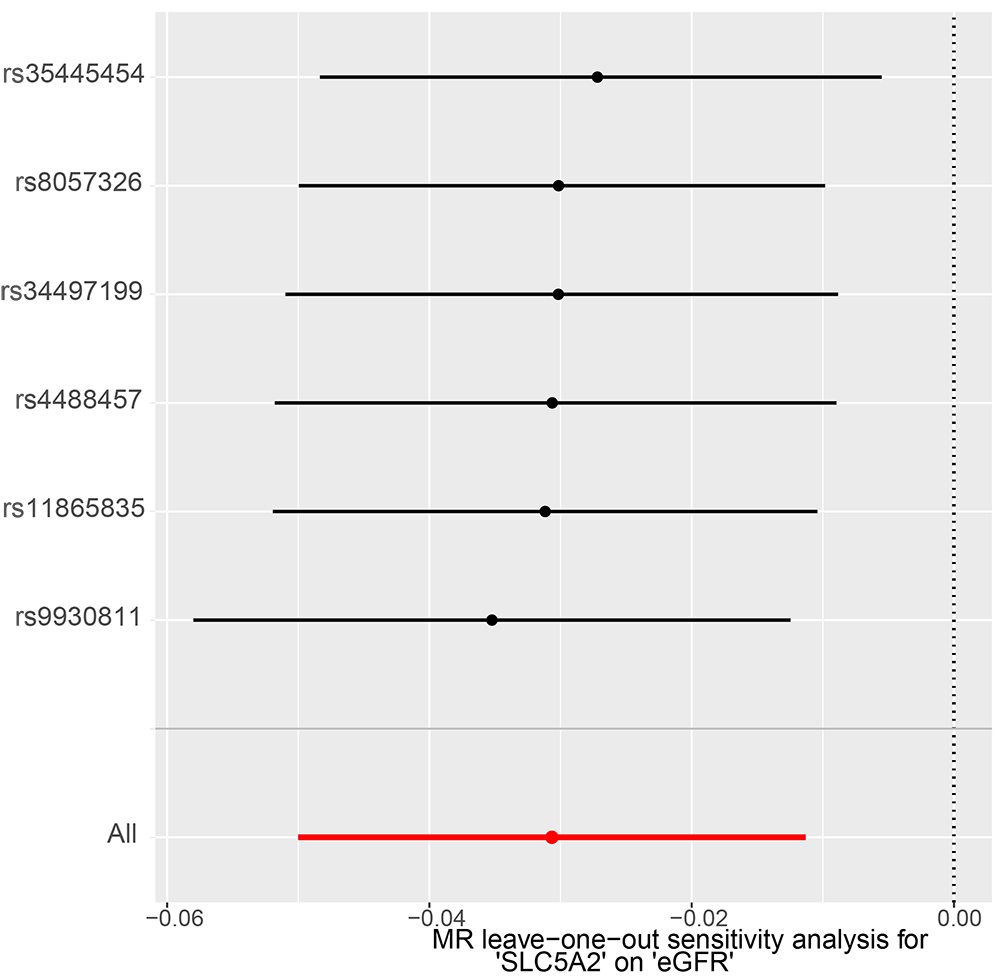

Supplement: Supplementary file 7 [file Image6.tif]
